# The Benefit and Harm of an Implantable Cardiac Defibrillator in a Patient with Heart Failure: A Case Report and Literature Review

**DOI:** 10.3390/reports8010030

**Published:** 2025-03-12

**Authors:** Mihai Grigore, Andreea-Maria Grigore, Traian-Vasile Constantin, Viorel Jinga, Adriana-Mihaela Ilieșiu

**Affiliations:** 1 Cardio-Thoracic Department, Carol Davila University of Medicine and Pharmacy, 021021 Bucharest, Romania; mihai.grigore@drd.umfcd.ro (M.G.);; 2 Internal Medicine and Cardiology Department, “Prof. Th. Burghele” Clinical Hospital, 050653 Bucharest, Romania; 3 Cardiology Department, Colentina Clinical Hospital, 020125 Bucharest, Romania; 4 Department of Urology, Carol Davila University of Medicine and Pharmacy, 8 Eroii Sanitari Blvd., 050474 Bucharest, Romania; 5 Academy of Romanian Scientists, 3 Ilfov, 050085 Bucharest, Romania

**Keywords:** implantable cardioverter-defibrillator, infective endocarditis, heart failure, renal tumor

## Abstract

**Background and Clinical Significance**: Infective endocarditis (IE) in patients with cardiac implantable electronic devices (CIED-IE) is a severe condition with high mortality and increasing prevalence. **Case Presentation**: A 50-year-old man with diabetes, idiopathic dilated cardiomyopathy, and a dual-chamber implantable cardioverter-defibrillator (ICD) for secondary prevention of sudden cardiac death (SCD) presented with fever and peripheral arthritis. Initially evaluated for rheumatic disease, IE was ruled out at an initial cardiac evaluation. A subsequent computed tomography scan revealed a renal tumor, leading to transfer to the urology department. The patient was later evaluated in our cardiology department, where transthoracic and transesophageal echocardiography identified lead vegetations. Blood cultures and serologic tests remained negative. Empirical antibiotic therapy was initiated, and percutaneous ICD lead extraction was performed, with cultures remaining negative. After six weeks of antibiotic therapy the patient had a favorable outcome, then a subcutaneous ICD (S-ICD) was implanted three months later for SCD prevention. The renal tumor required nephrectomy, confirming clear cell renal carcinoma. **Conclusions**: This case highlights the diagnostic and therapeutic challenges of blood culture-negative CIED-IE and underscores the importance of a multidisciplinary approach in complex cases.

## 1. Introduction and Clinical Significance

### 1.1. Literature Review

The incidence of infective endocarditis (IE) associated with cardiac implantable electronic devices (CIED-IE) has risen alongside the increasing number of these devices being implanted [1]. CIED-IE represents a severe complication with significant mortality, emphasizing the critical need for prevention, accurate diagnosis, and effective management in clinical practice [1].

CIED-IE typically arise from local infections caused by the patient’s endogenous bacterial flora during device implantation, despite adherence to rigorous preoperative protocols. Less commonly, bacteremia acts as the source of infection. The primary pathogens associated with these infections include coagulase-negative staphylococci (CoNS) in pocket infections and Staphylococcus aureus or CoNS in bacteremia cases [2,3]. Oral streptococci are also implicated, particularly in cases where IE develops long after device implantation [3]. According to the International Collaboration on Endocarditis-Prospective Cohort Study (ICE-PCS), the most common causative microorganisms in IE are Staphylococcus aureus (31%), followed by oral streptococci (17%), and CoNS (11%) [3].

Diagnosing CIED-IE is challenging due to the limited specificity of traditional criteria, such as the Duke criteria. Patients with CIED-IE typically present with non-specific symptoms, such as fever, chills, or signs of embolic phenomena [1].

The clinical presentation and diagnosis of IE can be complicated by the occurrence of osteoarticular manifestations. A systematic review analyzed the incidence of secondary osteoarticular infections in these patients, with reported rates ranging from 2.5% to 10.6%. The most recent and relevant case series suggest that approximately 3.5–3.9% of patients with IE develop synchronous septic arthritis [4].

The diagnosis of CIED-IE is based on clinical presentation, imaging findings—such as vegetations observed on leads—or advanced imaging techniques like fluorodeoxyglucose positron emission tomography (FDG-PET) scans. Infections are classified as localized (affecting the superficial incision site or device pocket) or systemic (involving the heart valves or leads, with or without vegetations) [1].

Serologic testing plays a crucial role in diagnosing endocarditis caused by fastidious or non-cultivable organisms, such as *Coxiella burnetii* and *Bartonella* species. *Coxiella burnetii* serology is the most validated test, with anti-phase I IgG titers ≥1:800 serving as a major Duke criterion for IE diagnosis. Bartonella endocarditis is also frequently diagnosed serologically, though the test results may be difficult to assess due to the cross-reactivity with Chlamydia/Chlamydophila species. Despite improving the diagnostic accuracy, the serologic test is not recommended due to the risk of false positive results [5,6].

Several factors increase the risk of CIED-IE, including patient-related factors (e.g., diabetes mellitus, end-stage renal disease, and heart failure), procedure-related factors (e.g., device generator replacement), and device-related factors [2]. The PADIT (Prevention of Arrhythmia Device Infection Trial) score, which includes prior procedures, age, renal insufficiency, immunosuppression, and type of procedure, has been validated in large-scale studies as a reliable tool for infection risk stratification [1].

Complete extraction of the CIEDs is recommended for all patients with confirmed lead infections, as conservative management has been linked to higher mortality rates [7]. The lead extraction should be performed promptly, ideally within the first few days of hospitalization, as this is associated with better outcomes [7]. A percutaneous approach is generally preferred, though it requires specialized expertise. If large vegetations are present, they may be aspirated percutaneously before lead removal to minimize the risk of embolization [8]. Surgical extraction should be considered for large vegetations (greater than 20 mm) if aspiration is not feasible or unsuccessful and is also preferred when there is associated valvular involvement requiring surgical repair [8,9].

There is no definitive evidence in the literature regarding the optimal timing for re-implantation of CIEDs [10]. However, it is generally recommended to wait at least 72 h after the extraction of CIEDs, once the inflammatory response has subsided and cultures are negative. If vegetations were present, re-implantation should be delayed until 2 weeks after negative blood cultures [11]. The patient’s risk of sudden death should be evaluated, and if deemed high, a wearable defibrillator may be considered as a temporary solution before re-implantation [1]. In cases with a high risk of reinfection, a subcutaneous ICD (S-ICD) may be considered. Recent guidelines support the non-inferiority of S-ICD for patients who do not need pacing for bradycardia, anti-tachycardia pacing, or cardiac resynchronization [1].

Preventive measures are crucial in minimizing the risk of CIED-IE. These include preoperative prophylactic antibiotics and strict aseptic techniques during device implantation [1]. Prophylactic antibiotics are generally unnecessary for dental, respiratory, gastrointestinal, or genitourinary procedures but are essential before device implantation [1].

There are data that confirms the efficacy of an antibacterial envelope in preventing CIED pocket infections, particularly in high-risk patients identified by the PADIT score. Selective use based on risk stratification may improve cost-effectiveness, warranting further prospective research in high-risk populations [12].

### 1.2. EI and Cancer

The relationship between cancer and IE is complex. In a study, 30% of patients with EI and cancer had blood culture–negative endocarditis, consistent with previous findings of a 42% prevalence of IE in cancer patients. Early antibiotic use, non-infectious vegetations, and limited PCR-based pathogen identification are possible explanations. Diagnostic yield may improve using reverse transcription-polymerase chain reaction, valvular biopsies, serology, and the evaluation for autoimmune or thrombotic causes. The non-bacterial thrombotic endocarditis (NBTE) is linked to embolic events in solid tumors, commonly seen in advanced malignancy. Differentiating true culture-negative IE from NBTE remains challenging. Advanced imaging, including FDG-PET/CT and labeled white blood cell scintigraphy (WBCS) improves the diagnosis, particularly in the prosthetic valve and CIED-IE [13,14,15,16,17,18,19,20].

The main mechanisms of NBTE are endothelial injury, hypercoagulability, hypoxia, and immune complex deposition leading to occurrence of sterile vegetations. Although NBTE predominantly affects native or prosthetic valves, the device leads could rarely be involved, emphasizing the need for thorough echocardiographic assessment [6,21].

In summary, the management of CIED-IE requires a comprehensive approach that includes preventive strategies, prompt diagnosis, and timely treatment to optimize patient outcomes. Re-implantation decisions must be carefully planned, taking into account the patient’s clinical status and risk of reinfection.

## 2. Case Presentation

A 50-year-old patient with peripheral arthritis and arthralgias affecting the hands, knees, and ankles, persistent fever, and diaphoresis, was admitted to the rheumatology department with clinical suspicion of rheumatoid arthritis.

The patient’s medical history included a ten-year history of type 2 diabetes mellitus managed with oral antidiabetic drugs, and idiopathic dilated cardiomyopathy (IDCM) with severe systolic dysfunction (left ventricular ejection fraction [LVEF] 25%). The diagnosis of IDCM was established five years ago after electrical cardioversion of an episode of ventricular tachycardia, based on clinical evaluation and echocardiographic findings. A bicameral ICD had been implanted for secondary prevention of sudden cardiac death (SCD). The patient had normal coronary angiography, without atherosclerotic epicardial lesions, and had no family history of heart disease. Cardiac MRI and genetic testing were not performed. Chronic treatment for heart failure with reduced ejection fraction (HFrEF) included metoprolol succinate 150 mg OD, perindopril 5 mg OD, spironolactone 25 mg OD, furosemide 40 mg OD, and atorvastatin 40 mg OD for dyslipidemia. It is of note that one month before, the patient had undergone periosteal dental procedures without antibiotic prophylaxis.

**Figure 1:**
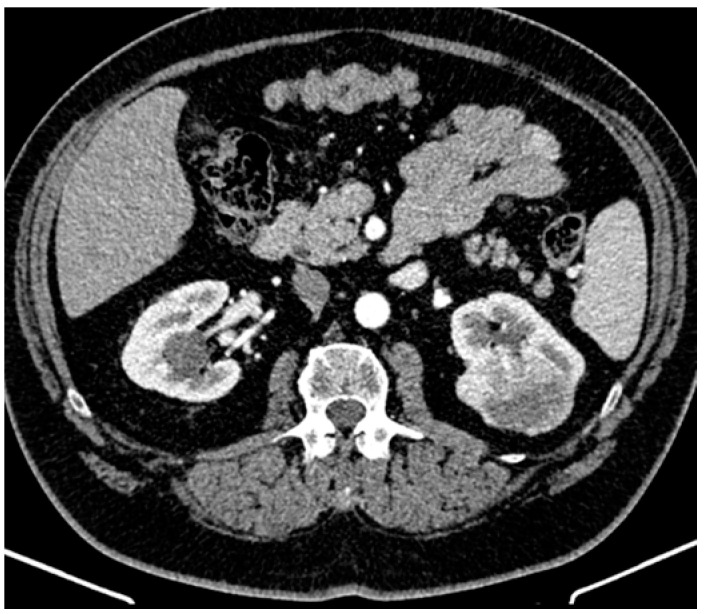
Abdominal computed tomography revealed a left kidney mass extending into the perirenal fat up to the renal fascia without invading it, involving the upper calyceal groups.

The patient did not meet the criteria for rheumatoid arthritis based on the 2010 ACR/EULAR Rheumatoid Arthritis Classification Criteria given the negative serology for rheumatoid factor and cyclic citrullinated peptide. Other autoimmune diseases were excluded as well. The presence of systemic symptoms (fever and chills), mild leukocytosis, and elevated inflammatory markers in a diabetic with an ICD was highly suggestive of IE and the patient was transferred to the cardiology department of another hospital for further evaluation. The diagnosis of IDCM was confirmed, but the transthoracic (TTE) and transesophageal (TOE) echocardiography did not reveal vegetations. Three separately collected blood samples of 10 mL each were obtained within 24 h, prior to antibiotic initiation, during fever episodes, following strict aseptic and antiseptic protocols. Subsequently, daily blood samples of 10 mL were collected during fever episodes, with antimicrobial-neutralizing agents. All blood cultures were monitored for up to three weeks. Due to the persistence of infectious symptoms, empirical antibiotic therapy was started. To identify a potential infectious collection, a thoracic and abdominal computed tomography (CT) was performed, revealing a mass at the upper pole of the left kidney suggestive for a renal tumor (Figure 1). The patient was referred to the urology department of our hospital. The abdominal magnetic resonance imaging (MRI) confirmed a renal tumor of 6.3 cm diameter, with contrast enhancement and the presence of a “washing out” phenomena.

Due to the persistence of fever (seven days), arthralgias, and arthritis, the patient was subsequently referred after three days to our cardiology department for cardiovascular re-evaluation and appropriate treatment.

**Figure 2:**
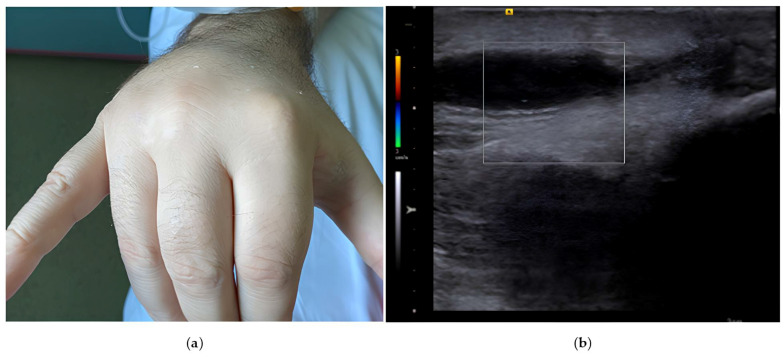
(**a**) Swelling in the dorsal region of the right hand, with flexion deformities of fingers II–IV and extension of the fifth finger and thumb. (**b**) Ultrasound examination of the hand showing an anechoic area with no Doppler signal adjacent to the extensor tendons, suggestive of tenosynovitis.

On clinical examination, the patient was febrile (39 °C), without signs of congestion, with painful mobilization of the right wrist joint, painful swelling of the dorsal region of the right hand, and of the right metacarpophalangeal joints, and flexion deformities of fingers II–IV and extension of the fifth finger and thumb (Figure 2). There was no clinical evidence of infection at the device pocket site.

**Figure 3:**
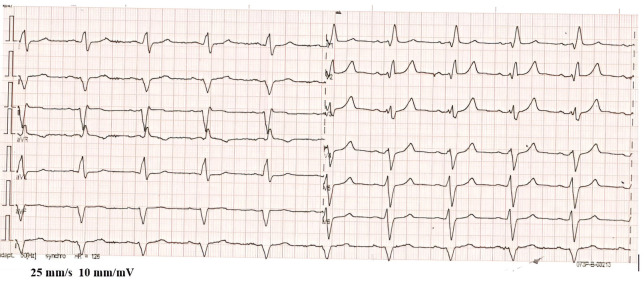
ECG: sinus rhythm, left-axis deviation, and right bundle branch block.

On an electrocardiogram (ECG) the patient had sinus rhythm and bifascicular block (left anterior hemiblock and the right bundle branch block (Figure 3).

Laboratory data showed leukocytosis 17,000 cells/µL with neutrophilia, mild normochromic normocytic anemia with hemoglobin 11.2 g/dL, marked inflammatory syndrome with elevated C-reactive protein (CRP) of 180 mg/L, and an erythrocyte sedimentation rate (ESR) of 87 mm/h. NT-proBNP was increased to 1030 pg/mL, and Hs Troponin I, glycemia, and the renal function were within normal limits.

**Figure 4:**
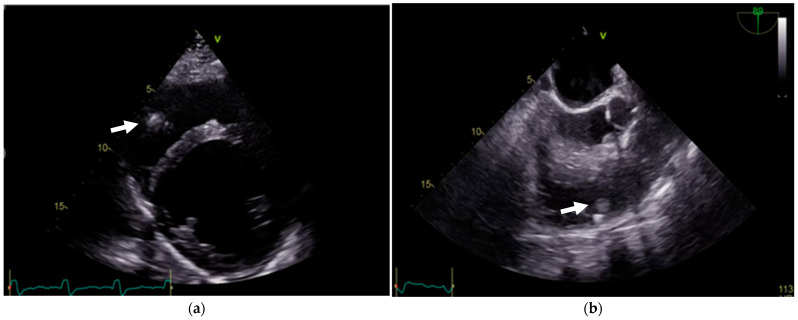
(**a**) TTE showing a mobile vegetation (arrows) attached to the ICD lead in the right ventricle. (**b**) TEE confirming two additional vegetations attached to the ICD lead.

A TTE examination revealed a left ventricular (LV) end-diastolic dimension of 59 mm, a left ventricular end-systolic dimension of 49 mm, and a biplane LVEF of 25%. LV filling pressure was normal. The left atrium volume indexed was 41 mL/m^2^. Mild secondary mitral regurgitation was observed. The right ventricle (RV) had a basal dimension of 38 mm, and the tricuspid annular plane systolic excursion was 19 mm. The right atrium volume indexed was 28 mL/m^2^, with mild secondary tricuspid regurgitation. The estimated systolic pulmonary artery pressure was 37 mm Hg. A mobile vegetation approximately 10 mm in size was detected, attached to the ICD lead in the right ventricle. At TEE, another two additional vegetations attached to the ICD lead were identified (Figure 4).

The thoracic CT revealed septic pulmonary embolism. The spleen size was normal on abdominal ultrasound (12 cm).

Repeat blood cultures were negative, including those targeting atypical pathogens.

Serologic testing for Coxiella burnetii and Bartonella species yielded negative results.

Based on the Duke criteria, the diagnosis of CIED-IE with negative blood cultures was established.

Following the recommendation of the infectious disease specialist, empirical antibiotic therapy was initiated targeting methicillin-resistant Staphylococcus aureus (MRSA) and Gram-negative bacteria, consisting of vancomycin (30 mg/kg/day), gentamicin (3 mg/kg/day), and meropenem (1 g every 8 h), with no clinical improvement.

**Figure 5:**
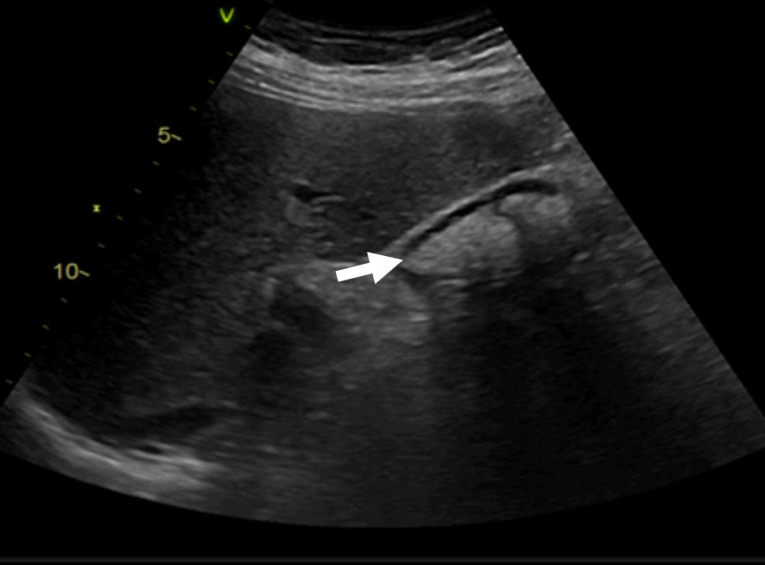
Abdominal ultrasound showing biliary sludge (arrow) associated with ceftriaxone therapy.

The antibiotic regimen was changed to linezolid (600 mg BID), ceftriaxone (2 g OD), and doxycycline (100 mg BID). Minimal clinical improvement occurred, but the following significant side effects of antibiotic therapy occurred: bone marrow aplasia resulting in pancytopenia due to linezolid and mildly symptomatic biliary sludge after ceftriaxone treatment (Figure 5).

Despite antibiotic treatment, the inflammatory markers showed limited improvement during the course of hospitalization. On day 3 of hospitalization, the leukocyte count was 16,500 cells/µL, CRP was 150 mg/L, and the ESR was 82 mm/h.

The patient had a strong indication for urgent complete extraction of the CIED system. He was transferred on day 3 of hospitalization to a center with expertise, where the complete CIED system was successfully removed using transvenous lead extraction by simple traction. ICD interrogation confirmed that the patient did not require cardiac pacing. The patient was then sent back to our clinic for further monitoring and treatment.

The patient’s HFrEF management was optimized according to the latest European Society of Cardiology guidelines. Sacubitril/valsartan was titrated to the maximum dose (97/103 mg BID), metoprolol succinate increased to 200 mg OD, spironolactone to 50 mg OD, and dapagliflozin (10 mg OD) was added. Due to the high risk of deep vein thrombosis (8 points on the Padua Prediction Score for risk of VTE), the patient received prophylactic anticoagulation with enoxaparin 40 mg once daily.

**Figure 6:**
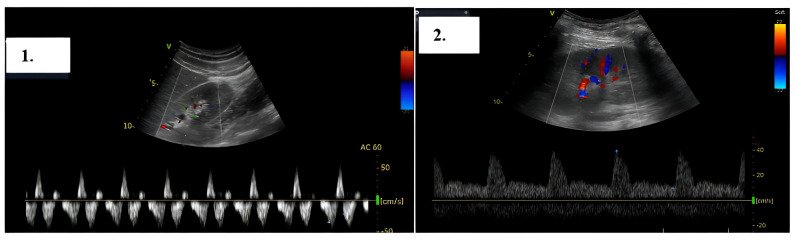
Right intrarenal-venous flow. In the left image 1, discontinuous intrarenal venous flow with S wave < D wave is observed, while in the right image 2, continuous intrarenal venous flow is seen.

During the hospitalization, the association of severe infection with the myocardium and the saline solution infusion for the antibiotic treatment led to heart failure decompensation with subclinical congestion. The patient was monitored for signs of congestion using systemic ultrasound (inferior vena cava diameter, hepatic veins flow, and renal venous flow) on a weekly basis. Changes in renal venous flow during ultrasound monitoring were suggestive of subclinical congestion. Diuretic therapy with furosemide was initiated with an improvement of/normalized renal venous flow profile, indicative of decongestion (Figure 6).

The antibiotic treatment with Ceftriaxone 2 g OD and Doxycycline 100 mg BID continued until 6 weeks. The patient became afebrile, with significant clinical improvement and the remission of infection and inflammation. The signs of tenosynovitis and osteoarthritis disappeared. Pathological examination confirmed vegetation on the ICD lead. The extracted ICD lead was inoculated in culture media and monitored for seven days, but no microbial growth was observed.

After the successful ICD lead extraction and completion of a six-week antibiotic therapy, laboratory data showed a significant reduction in inflammatory markers. By the end of the treatment, the leukocyte count had normalized to 8000 cells/µL, CRP had decreased to 5 mg/L, and ESR dropped to 22 mm/h.

After the treatment of CIED-IE with antibiotics associated with ICD lead extraction, and the improvement due to the HfrEF therapy, the patient became asymptomatic, without fever and with improvement of the left ventricular (LVEF of 45%). The arthritis and arthralgias subsided, and the inflammatory markers were in the normal limits.

**Figure 7:**
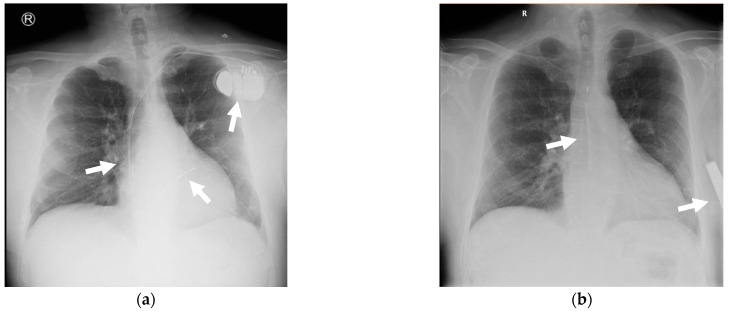
Chest X-ray: (**a**) on the left side (at admission), a bicameral ICD is visible, with arrows indicating the generator located in the left subclavicular region, the leads situated in the right atrium and right ventricle, respectively. On the right side (**b**) after S-ICD implantation, the chest X-ray shows arrows indicating the S-ICD electrode and the S-ICD generator.

After three months, to reduce the risk of recurrent CIED-IE and in the absence of indication for cardiac resynchronization therapy or pacing, an S-ICD was implanted (Figure 7). The timing of the S-ICD implantation was not based on immediate medical necessity, but rather on the availability of resources, as S-ICD is a relatively new therapy, and its use was contingent on its accessibility at the time.

The patient initially postponed the nephrology recommendation and underwent left nephrectomy after one year. The histopathological diagnosis was clear cell renal carcinoma without perirenal invasion.

## 3. Discussion

This case underscores the diagnostic and therapeutic complexity of CIED-IE, particularly in the absence of identified pathogens. Despite multiple blood cultures taken during febrile episodes and after discontinuation antibiotic therapy, all cultures remained negative. The diagnostic challenge of culture-negative infective endocarditis is well-documented in the ESC (European Society of Cardiology) guidelines for the management of endocarditis, which emphasize the importance of performing blood cultures prior to antibiotic administration and necessitating specialized culturing techniques or molecular approaches, such as 16S and 18S ribosomal ribonucleic acid (RNA) sequencing [1].

In this patient, the pathological analysis of the vegetation excised from the device lead would have provided critical diagnostic insights. However, despite histopathological examination, the microorganism responsible for the infection could not be identified. According to the ESC guidelines for the management of endocarditis, histopathological examination of excised tissue remains the gold standard for diagnosing IE as it facilitates the identification of microorganisms through specific stains and molecular techniques [1].

The patient’s clinical presentation further contributed to the diagnostic difficulty. Peripheral joint involvement, including tenosynovitis of the right hand, is an uncommon manifestation of IE [22]. This atypical feature, combined with the incidental finding of a renal tumor (initially suspected as the source of systemic inflammation), delayed the diagnosis. Renal carcinoma itself can present with fever, night sweats, and weight loss, mimicking the inflammatory profile of IE [23,24].

Another diagnostic consideration in this case was the dental procedure performed before symptom onset. Subacute evolution and the patient’s history suggested a possible oral streptococcal origin, yet no pathogens were isolated. ESC guidelines highlight the diagnostic challenge of systemic CIED infections without local signs, where non-specific symptoms such as fever, chills, and night sweats can lead to delayed recognition [1].

The echocardiographic findings also contributed to the complexity. The appearance of the vegetation could have been mistaken for a cardiac fibroelastoma, which originates from native cardiac structures and not implanted devices. Such imaging findings, coupled with negative blood cultures, necessitate a multidisciplinary approach for accurate diagnosis and management.

Regarding patient management, this case raises important questions about antibiotic prophylaxis before dental procedures in patients with CIEDs. Current international guidelines do not mandate prophylaxis in these cases, though further studies are needed to evaluate its utility in reducing the risk of CIED-IE [1].

The patient’s predisposing factors, including diabetes mellitus, heart failure, and renal carcinoma, likely contributed to both the onset of IE and the decompensation of heart failure. Incomplete infection control, compounded by saline infusions, may have exacerbated the patient’s volume status, leading to heart failure decompensation. Novel methods for assessing congestion and decongestion, such as renal venous flow analysis, could provide valuable insights into the hemodynamic changes associated with CIED-IE [25].

Finally, the decision to re-implant a subcutaneous implantable cardioverter-defibrillator (S-ICD) after complete hardware removal was guided by clinical consensus [1,26]. ESC guidelines for the management of endocarditis recommend delaying re-implantation until systemic and local signs of infection resolve, with blood cultures remaining negative for at least 72 h [1]. Although no randomized trials guide the timing of re-implantation, individualized decisions are crucial. S-ICDs represent a safe and effective alternative to transvenous systems, especially in patients with prior infections, offering comparable outcomes in terms of efficacy and safety [26].

Renal cell carcinoma is the third most common urogenital cancer, with clear cell renal cell carcinoma accounting for 75–80% of cases and being associated with poorer survival compared to papillary and chromophobe subtypes. Curative treatment for localized clear cell renal cell carcinoma involves radical or partial nephrectomy, while small tumors may be treated with radiofrequency ablation. Lymph node dissection is generally reserved for cases with suspected metastases. The patient requires regular imaging follow-up, including thoracic and abdominal CT, for at least five years post-surgery to enable early detection of disease progression [23,24].

This case underscores the importance of a multidisciplinary approach, involving oncologists, nephrologists, urologists, and cardiologists, to ensure personalized care, monitor progression, and optimize long-term outcomes.

## 4. Conclusions

This case highlights the diagnostic and therapeutic challenges of infective endocarditis affecting cardiac implantable electronic devices in systemic CIED infections with persistent negative blood culture, in a patient with multiple co-morbidities, including a kidney cancer discovered incidentally. The atypical manifestations, such as inflammatory joint involvement, and the need for repeating echocardiography within days in case of initially negative examination, but having a high clinical suspicion of CIED-IE, can delay the diagnosis.

Due to uncontrolled infection under antimicrobial treatment alone, lead removal is mandatory using percutaneous extraction as the preferred procedure, but it requires centers with expertise. The successful use of an S-ICD after device removal demonstrates a safe alternative in patients at high risk of recurrent infections.

## Data Availability

Data used in this study may be provided by the authors upon reasonable request due to privacy concern.

## Abbreviations

The following abbreviations are used in this manuscript:

CIED-IE	Infective endocarditis associated with cardiac implantable electronic devices
ICD	Implantable Cardiac Defibrillator
S-ICD	Subcutaneous Implantable Cardioverter-Defibrillator
CoNS	Coagulase-negative staphylococci
OD	Once a day
MRI	Magnetic Resonance Imaging
CT	Computerized Tomography
MRSA	Methicillin-resistant Staphylococcus aureus
HFrEF	Heart failure with reduced ejection fraction
ISUP	International Society of Urological Pathology
